# QTL mapping of morphological characteristics that correlated to drought tolerance in St. Augustinegrass

**DOI:** 10.1371/journal.pone.0268004

**Published:** 2022-05-02

**Authors:** Xingwang Yu, Nicolas A. H. Lara, Esdras M. Carbajal, Susana R. Milla-Lewis

**Affiliations:** Department of Crop and Soil Sciences, North Carolina State University, Raleigh, North Carolina, United States of America; New South Wales Department of Primary Industries, AUSTRALIA

## Abstract

St. Augustinegrass is a warm-season grass species widely utilized as turf in the southeastern U.S. It shows significant variation in plant growth and morphological characteristics, some of which are potentially associated with drought tolerance. However, the genetic basis of these variations is not well understood. Detecting quantitative trait loci (QTL) associated with morphological traits will provide a foundation for the application of genetic and molecular breeding in St. Augustinegrass. In this study, we report QTL associated with morphological traits, including leaf blade width (LW), leaf blade length (LL), canopy density (CD), and shoot growth orientation (SGO) in a St. Augustinegrass ‘Raleigh’ x ‘Seville’ mapping population containing 115 F_1_ hybrids. Phenotypic data were collected from one greenhouse and two field trials. Single and joint trial analyses were performed, finding significant phenotypic variance among the hybrids for all traits. Interval mapping (IM) and multiple QTL method (MQM) analysis detected seven QTL for CD, four for LL, five for LW, and two for SGO, which were distributed on linkage groups RLG1, RLG9, SLG3, SLG7, SLG8 and SLG9. In addition, three genomic regions where QTL colocalized were identified on Raleigh LG1 and Seville LG3. One genomic region on Seville LG3 overlapped with two previously reported drought-related QTL for leaf relative water content (RWC) and percent green cover (GC). Several candidate genes related to plant development and drought stress response were identified within QTL intervals. The QTL identified in this study represent a first step in identifying genes controlling morphological traits that might accelerate progress in selection of St. Augustinegrass lines with lower water usage.

## Introduction

St. Augustinegrass (*Stenotaphrum secundatum* [Walt.] Kuntze) (*2n = 2x =* 18) is a widely used warm-season turfgrass that is well adapted to the southern United States and Gulf Coastal regions [1]. St. Augustinegrass is characterized by coarse-textured leaf blades, rapid stolon growth, and good tolerance to shade stress, making this grass a popular option in the turf market in the southern U.S. [2]. However, prolonged drought and water shortages have limited the availability and quality of water used for irrigating landscapes and lawns, which is one of the greatest challenges the turfgrass industry is facing. Thus, there is a critical need to develop St. Augustinegrass cultivars with improved water use efficiency to address these concerns, and research is needed to delineate the genetic control mechanisms of drought tolerance in St. Augustinegrass.

Evapotranspiration (ET) is often used to quantify turfgrass water use by the total amount of water lost for growth and transpiration (water loss from the leaf) plus the amount of water lost from the soil surface (evaporation) [3, 4]. Evapotranspiration rates vary across different turfgrass species and cultivars and are often affected by environmental conditions, including temperature, wind, solar radiation, relative humidity, and soil moisture [5]. Beyond that, turfgrass water use is also affected by plant growth and canopy characteristics. Kim and Beard (1988) suggested that low water use in well-watered turfgrass may be associated with (i) high canopy resistance to ET, which combines characteristics such as high shoot and leaf density and a more horizontal leaf and shoot orientation, and (ii) low leaf area components including a narrow leaf width and slow vertical leaf extension rates [6]. Turfgrass that possesses high shoot and leaf density and a substantial horizontal leaf orientation may increase resistance to the water vapor movement through the canopy. Low leaf area components reduce total leaf area and water loss from transpiration surfaces [7]. Huang (2008) suggested that grass species with low water use may possess at least one of the combined characteristics of slow vertical growth, prostrate growth pattern, and dense canopy [8]. However, the genetic architecture of these leaf morphological and canopy characteristics is not well understood in St. Augustinegrass.

Recently, substantial genetics and genomics information has been generated in St. Augustinegrass by taking advantage of next-generation DNA sequencing technology, which has allowed genetic analysis and QTL mapping to be higher efficient. Yu et al. (2018) developed high-density linkage maps containing 2871 single nucleotide polymorphism (SNP) markers from a ‘Raleigh x Seville’ F_1_ population in St. Augustinegrass [9]. This population later proved to be segregating for drought tolerance in both greenhouses and field trials, and QTL controlling several drought-related physiological traits were identified [10]. Considering the turfgrass water use also could be affected by plant growth and canopy characteristics, we intend to further characterize the morphological traits of ‘Raleigh x Seville’ population, and mapping QTL related to the interest traits, which will shed light on the genetic control of water usage and its potential application in marker-assisted selection in St. Augustinegrass. Thus, the objectives of this study include (i) evaluating variance of morphological traits in a ‘Raleigh x Seville’ F_1_ population in both greenhouses and field trials and (ii) identifying QTL associated with morphological and canopy traits that might have a role in drought tolerance.

## Materials and methods

### Mapping population

The ‘Raleigh’ x ‘Seville’ F_1_ mapping population of St. Augustinegrass used in this study was previously developed by Kimball et al. (2018) and contains 115 hybrids [11]. The SNP-based high-density linkage maps derived from this ‘Raleigh x Seville’ population were developed by Yu et al. (2018) [9]. In general, two parental maps were created using pseudo-testcross method that containing nine linkage groups for each parent, which correspond to the nine chromosomes for diploid St. Augustinegrass, named RLG1-RLG9 for the Raleigh map and SLG1-SLG9 for the Seville map. For QTL analysis, clones of all 115 F_1_ individuals and parental lines were planted in randomized complete block designs with three replicates at the North Carolina State University Greenhouses (GH; Raleigh, NC), the Lake Wheeler Turf Field Laboratory (TFL; Raleigh, NC) and the Sandhills Research Station (SRS; Jackson Springs, NC).

### Phenotypic analysis

Plants in the greenhouse trial were vegetatively propagated in 15 cm diameter by 11 cm deep pots filled with a mix of sand and Fafard potting mix (Conrad Fafard Inc, Agawam, MA). Plants were established for ten weeks to allow sufficient growth to form a uniform canopy. The TFL and SRS field trials were planted from eight 4-inch plugs in 0.91 m × 0.91 m plots with 0.46 m alleys in between. Plots were mowed weekly at a height of 6.35 cm, irrigated, fertilized and pesticide applied according to recommended practices for NC [11]. Three morphological traits related to water usage were recorded across the three experimental trials including leaf blade width (LW), leaf blade length (LL), and canopy density (CD). Fully expanded leaves on similar maturity were collected between mowing interval for measurement. For LW and LL a digital caliper was used to measure five fully expanded leaf samples per pot/plot. Leaf width was measured at the widest point of the leaf (midpoint), and LL was measured as the length of the blade from the collar region to the leaf tip [7]. Canopy density was visually rated according to the National Turfgrass Evaluation Program’s (NTEP) guidelines on scale of 1–9 where 9 indicates maximum density [12]. In addition, shoot growth orientation (SGO) was collected only from the greenhouse trial and was estimated visually on a scale of 1 to 9, with 1 being entirely vertical and 9 being entirely horizontal [6].

Morphological data were analyzed using the GLM procedure in SAS (SAS Institute, Cary, NC). Correlation analysis was performed using the CORR procedure in SAS (SAS Institute, Cary, NC). Least square means (LSmeans) were calculated using PROC GLM to obtain the average value over three replicates, which were used for QTL analysis in each trial. In addition, QTL were identified across trials using “estimated” best linear unbiased predications (BLUP) of the set of genotypes evaluated. BLUP values were determined using the R package ‘lme4’ [13] following the model: *Y*_*ijk*_ = μ + *E*_*i*_ + *R(E)*_*ji*_ + *G*_*k*_ + *GE*_*ki*_ + ε, where *E*_*i*_ = effect of trial *i*; *R(E)*_*ji*_ = effect of replicate *j* within trial *i*; *G*_*k*_ = effect of genotype *k*; *GE*_*ki*_ = effect of interaction between genotype *k* and trial *i*; ε = effect of error. All terms were considered as random except for the overall mean (μ). Broad-sense heritability (*H*^*2*^) was calculated according to *H*^*2*^ = Vg/(Vg + V_GE_/E + Ve/E_*_R), where Vg = variance for genotype; V_GE_ = variance for genotype-trial interaction; Ve = residual variance.

### QTL detection

QTL analysis was performed using the integrated two-way pseudo-testcross approach with MapQTL 6.0 [14], which was applied by analyzing data for each parental meiosis separately. Interval mapping (IM) and multiple QTL method (MQM) analysis were performed to detect significant associations between markers and phenotypic traits using a regression approach (S1 File). Genome-wide LOD thresholds (*p* < 0.05) were determined for each trait using a permutation test with 10,000 iterations. Regions with a LOD score above threshold values were considered as potential QTL intervals. QTL that overlapped in same regions were considered as colocalized QTL. In addition, the sequences flanking SNP markers within the QTL intervals were searched against the NCBI NR database to obtain the orthologous genes using the NCBI blastn tool with an e-value cutoff of 1 × 10^−5^ [15]. Gene annotation was conducted using the UniProt database to predict gene function in the QTL regions.

## Results

### Phenotypic trait analysis

**Fig 1:**
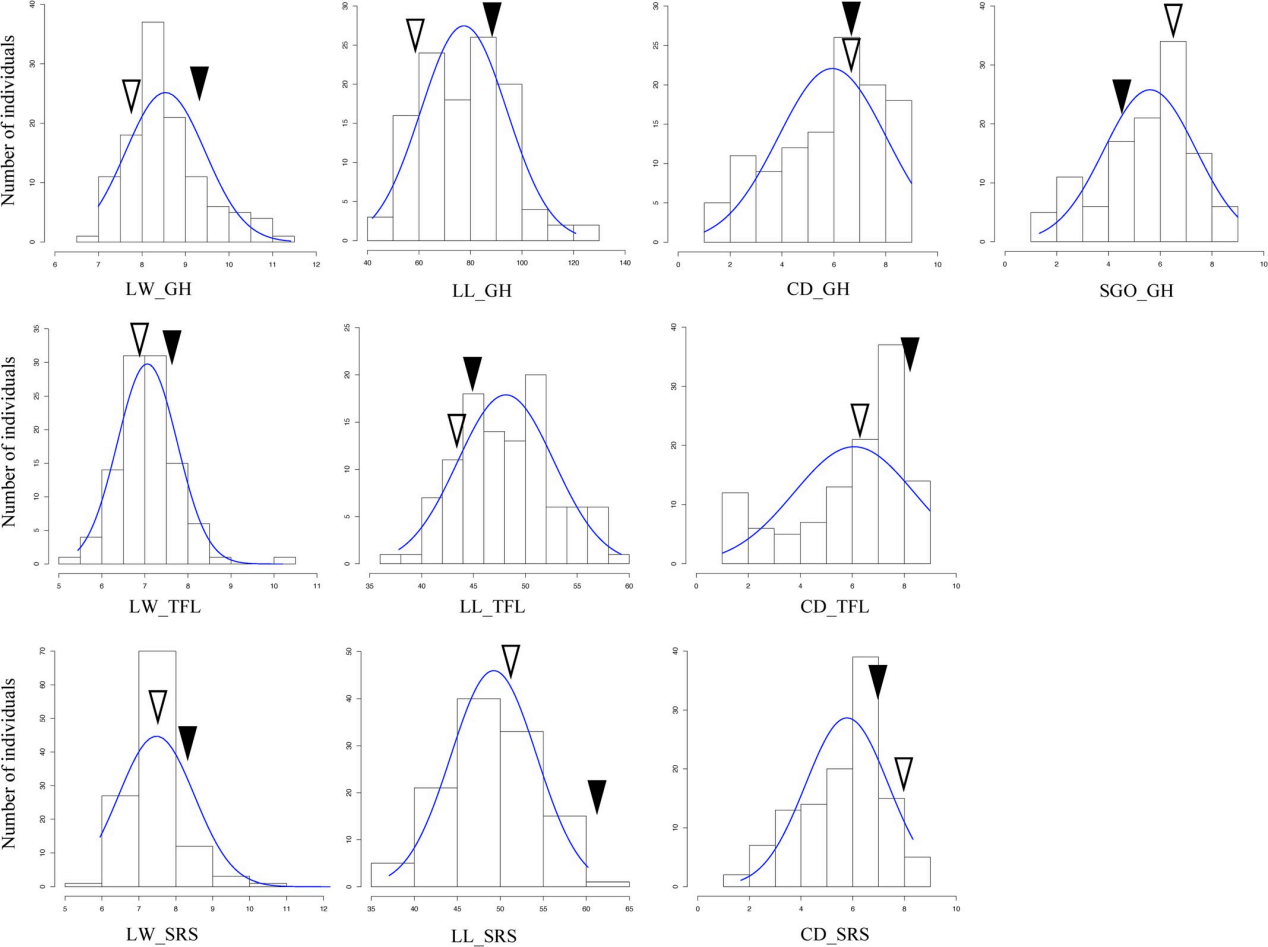
Distribution of leaf width (LW), leaf length (LL), canopy density (CD) and shoot growth orientation (SGO) for a St. Augustinegrass Raleigh x Seville F_1_ population evaluated in greenhouse (GH) and field (TFL and SRS) experiments. Solid triangle indicates value of Raleigh and white triangle indicates value of Seville.

The ‘Raleigh x Seville’ mapping population showed a wide range of phenotypic variation for all evaluated traits and in all independent trials (Table 1, Fig 1). Values for leaf width (LW) ranged from 6.99 to 11.42 mm at GH, 5.44 to 10.2 mm at TFL, and 5.95 to 15.57 mm at SRS. Leaf length (LL) ranged from 41.93 to 120.87 mm at GH, 37.8 to 59.26 mm at TFL, and 37.07 to 60.22 mm at SRS. Canopy density (CD) ranged from 1 to 9 at GH and TFL, and 1.67 to 8.33 at SRS. Shoot growth orientation (SGO) ranged from 1.33 to 9 at GH (Table 1). In addition, significant effects of genotype, location, and their interaction were observed for all morphological traits (Table 1). QTL analysis was conducted using both single location phenotypes and average values across locations.

**Table 1:** Phenotypic data and variance components for leaf width (LW), leaf length (LL), canopy density (CD) and shoot growth orientation (SGO) for a St. Augustinegrass Raleigh x Seville F_1_ population evaluated in greenhouse (GH) and field (Lake Wheeler Turf Field Laboratory, TFL and Sandhills Research Station, SRS) trials.

				Progeny value			Variance			Heritability (*H*^*2*^)
Trait	Trial	Raleigh	Seville	Min.	Mean	Max.	Genotype (G)	Location (L)	G x L	
**LW(mm)**	GH	9.21	7.81	6.99	8.54	11.42	< .0001	< .0001	< .0001	0.611
	TFL	7.59	6.91	5.44	7.06	10.20				
	SRS	8.29	7.53	5.95	7.48	15.57				
**LL (mm)**	GH	89.70	59.30	41.93	77.47	120.87	< .0001	< .001	< .0001	0.385
	TFL	45.49	44.42	37.80	48.12	59.26				
	SRS	61.10	51.82	37.07	49.24	60.22				
**CD**	GH	6.67	6.67	1.00	5.94	9.00	< .0001	< .0001	< .0001	0.645
	TFL	8.33	6.33	1.00	6.07	9.00				
	SRS	7.00	8.00	1.67	5.78	8.33				
**SGO**	GH	4.33	6.67	1.33	5.61	9.00	< .0001	NS	NS	0.950

* NS, not significant.

The distributions of LW, LL, CD and SGO were approximately normal, typical of quantitative inheritance (Fig 1). In general, parent Raleigh showed higher trait values for LW and LL in all trials, while Seville showed higher SGO values in GH. The two parents showed similar values for CD in GH, but Raleigh was higher at TFL and Seville was higher at SRS (Fig 1). Transgressive segregation occurred for all traits towards both maternal and paternal directions, but it was not uniform, meaning there were different proportions of transgressive segregants for different traits and in different locations (Fig 1).

### Correlation of morphological and drought tolerance traits

**Fig 2:**
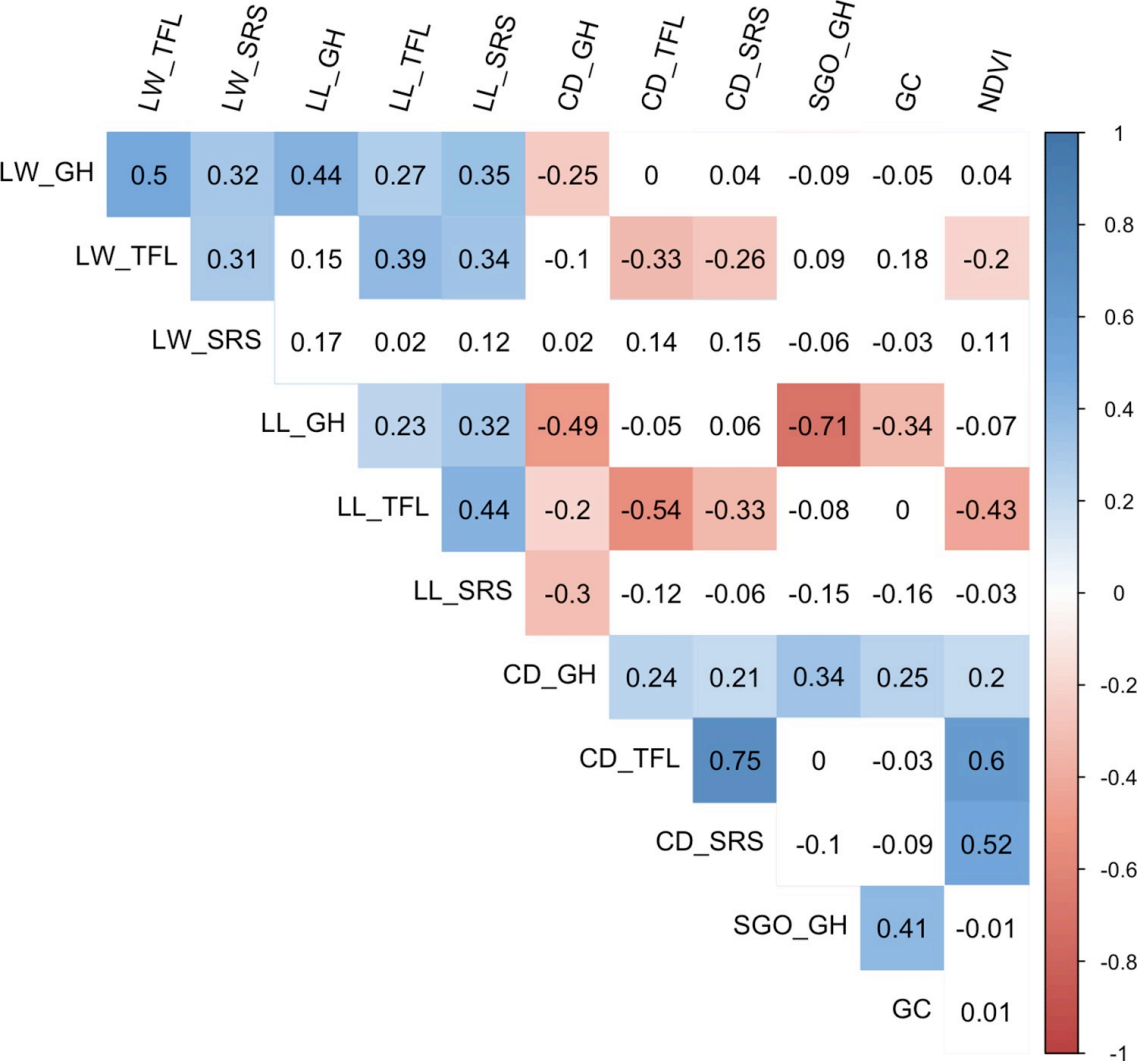
Correlation of morphological and drought traits for a St. Augustinegrass Raleigh x Seville F_1_ population evaluated in greenhouse (GH) and field (TFL and SRS). Morphological traits include leaf width (LW), leaf length (LL), canopy density (CD) and shoot growth orientation (SGO), while drought traits include percent green cover (GC) and normalized difference vegetation index (NDVI) reported in Yu et al. (2019) [10]. Blue color indicates positive correlation, while red color indicates negative correlation, with more intense colors for more extreme correlations. Correlations not significantly different from 0 are represented by a white box.

Pearson correlation coefficient analysis was conducted to determine the correlation among different traits and trials. All evaluated traits except SGO exhibited significant (*p* < 0.05) positive correlations among different trials (Fig 2). Among different traits, LW showed positive correlation with LL in all three trials, negative correlation with CD in GH and TFL, and negative correlation with SGO in GH. In addition, LL showed negative correlation with CD in all three trials, and negative correlation with SGO in GH. Lastly, SGO exhibited positive correlation with CD in GH (Fig 2).

We further estimated the correlation between morphological traits and the drought related traits previously reported in Yu et al. (2019) [10], which included green cover precent (GC) in greenhouse experiments and normalized difference vegetative index (NDVI) in field trials. In general, LW and LL showed negative correlations with drought related traits, while CD and SGO showed positive correlations (Fig 2). In the greenhouse trial, LL, CD and SGO showed significant correlation with GC, while LW was not significantly correlated with drought GC. In the TFL trial, all three traits (LL, LW and CD) showed significant correlation coefficients with drought NDVI. However, in the SRS trial, only CD showed significant positive correlation with NDVI, while correlations among NDVI and LW/LL were not significant (Fig 2).

### QTL detection for morphological traits

**Fig 3:**
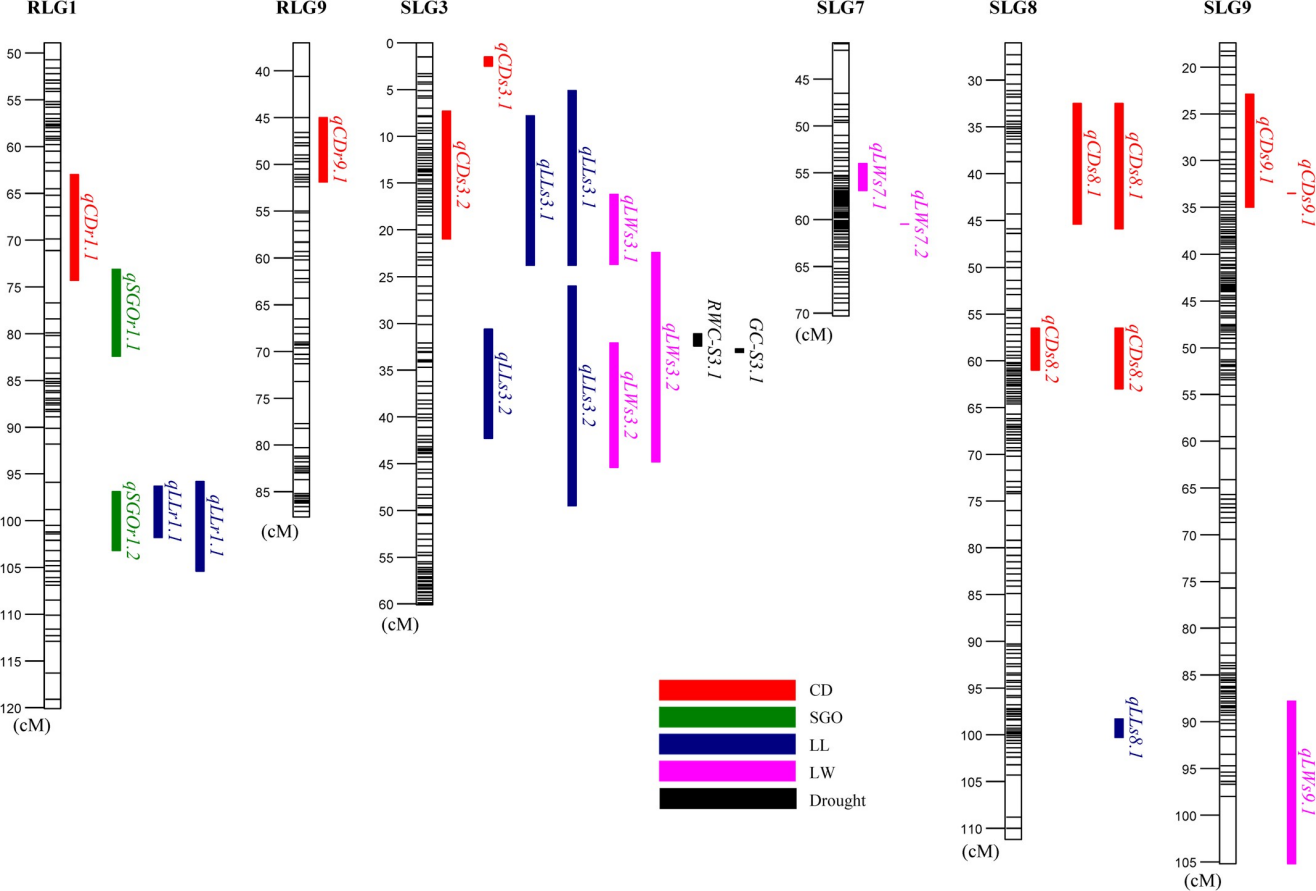
Distribution of quantitative trait loci (QTL) for morphological and canopy traits in a St. Augustinegrass Raleigh x Seville F_1_ population evaluated in greenhouse (GH) and field (TFL and SRS) trials. Morphological traits include leaf width (LW), leaf length (LL), canopy density (CD) and shoot growth orientation (SGO). QTL for drought traits including percent green cover (GC) and relative water content in leaves (RWC) were previously reported in Yu et al. (2019) [10].

Average trait values within and across trials (BLUP) were used to identify QTL on the parental linkage groups developed for the ‘Raleigh x Seville’ population [9]. Seven QTL were detected for CD, four for LL, five for LW, and two for SGO. These were distributed on linkage groups RLG1, RLG9, SLG3, SLG7, SLG8 and SLG9 (Table 2, Fig 3).

**Table 2:** Quantitative trait loci (QTL) found to have association with morphological traits in a St. Augustinegrass Raleigh x Seville F_1_ population evaluated in greenhouse (GH) and two field (TFL and SRS) trials.

QTL	Env.	LG	Peak Position (cM)	Interval (cM)	Nearest Marker	LOD	Explained Variance (%)	Additive Effect
**QTL for canopy density (CD)**								
*qCDr1*.*1*	GH	RLG1	67.41	63.02–74.27	SNP44527	4.68	17.2	-0.8601
*qCDs3*.*2*	GH	SLG3	16.87	7.25–20.98	SNP54623	4.24	15.7	-0.8233
*qCDs8*.*2*	GH	SLG8	56.63	56.49–60.98	SNP38207	3.75	14.1	-0.8017
*qCDs9*.*1*	SRS	SLG9	23.87	22.87–35.01	SNP17791	4.46	16.3	-0.6443
*qCDr9*.*1*	TFL	RLG9	51.91	44.99–51.91	SNP55871	4.61	16.9	0.9583
*qCDs8*.*1*	TFL	SLG8	44.35	32.53–45.35	SNP27553	3.93	14.6	-0.8837
*qCDs9*.*1*	TFL	SLG9	33.47		SNP32817	3.6	13.4	-0.8487
*qCDs3*.*1*	BLUP	SLG3	2.49	1.49–2.49	SNP34326	3.92	14.5	-0.3955
*qCDs8*.*1*	BLUP	SLG8	45.35	32.53–45.88	SNP43685	4.83	17.6	-0.4368
*qCDs8*.*2*	BLUP	SLG8	56.53	56.53–63.03	SNP38207	4.06	15	-0.3871
**QTL for leaf length (LL)**								
*qLLr1*.*1*	GH	RLG1	101.4	96.27–101.80	SNP30596	4.53	16.7	0.6884
*qLLs3*.*2*	GH	SLG3	33	30.63–42.31	SNP13076	4.51	16.6	0.6796
*qLLs3*.*1*	SRS	SLG3	20.78	7.77–23.78	SNP49435	4.45	16.3	2.0080
*qLLs8*.*1*	SRS	SLG8	98.3	98.30–100.28	SNP45500	3.77	14	1.9064
*qLLr1*.*1*	BLUP	RLG1	101.4	95.79–105.40	SNP30596	4.38	16.1	1.0851
*qLLs3*.*1*	BLUP	SLG3	20.78	5.14–23.78	SNP49435	4.88	17.8	1.1224
*qLLs3*.*2*	BLUP	SLG3	33	26.02–49.45	SNP13076	6.07	21.6	1.2440
**QTL for leaf width (LW)**								
*qLWs3*.*1*	GH	SLG3	22.55	16.23–23.69	SNP46773	5.43	19.7	0.4115
*qLWs3*.*2*	GH	SLG3	42.31	32.14–45.35	SNP47252	6.63	23.5	0.4505
*qLWs7*.*1*	GH	SLG7	55.4	54.04–56.88	SNP33371	4.69	17.3	0.3892
*qLWs7*.*2*	TFL	SLG7	60.54		SNP25872	3.51	13.1	0.2912
*qLWs3*.*2*	BLUP	SLG3	42.42	22.35–44.75	SNP47252	4.49	16.5	0.1623
*qLWs9*.*1*	BLUP	SLG9	103.03	87.83–105.18	SNP12790	4.74	17.3	0.1775
**QTL for shoot growth orientation (SGO)**								
*qSGOr1*.*1*	GH	RLG1	75.07	73.07–82.38	SNP2209	4.59	16.9	-0.8011
*qSGOr1*.*2*	GH	RLG1	101.4	96.87–103.19	SNP30596	5.21	19	-0.7790

* Env., environment. LG, linkage group. LOD, logarithm of odds.

For CD, there were three QTL identified in GH, one in SRS, three in TFL, and three QTL for the BLUP. These QTL explained from 13.4% to 17.6% of the variance. Among them, three QTL were identified in more than one environment, including *qCDs8*.*1* (TFL and BLUP), *qCDs8*.*2* (GH and BLUP) and *qCDs9*.*1* (SRS and TFL) (Table 2).

For LL, two QTL were detected in each GH and SRS and three for the BLUP, while no QTL was identified in TFL. These QTL were spread on RLG1, SLG3 and SLG8, explaining 14% to 21.6% of the phenotypic variance. Among them, *qLLr1*.*1* and *qLLs3*.*2* were detected in GH and BLUP, and *qLLs3*.*1* was detected in SRS and BLUP (Table 2).

For LW, three QTL were identified in GH, one in TFL, and two for the BLUP. Meanwhile, no QTL were identified in SRS. QTL for LW were located on SLG3, SLG7, and SLG9, explaining 13.1% - 23.5% of the phenotypic variance. There was only one QTL (*qLWs3*.*2*) identified in multiple environments (GH and BLUP) (Table 2).

QTL analysis for SGO included only GH data. Two QTL, located in different regions of RLG1 and explaining 16.9% and 19% of the phenotypic variance, were identified (Table 2).

### Colocalization of QTL

Following QTL analysis, several QTL for different traits were colocalized in the same genomic regions. On linkage group RLG1, QTL for SGO (*qSGOr1*.*2*) and LL (*qLLr1*.*1*) overlapped in the same region (96.27–101.80 cM) (Table 2, Fig 3). Notably, there were two colocalized regions on linkage group SLG3. In the first region (5.14–23.78), QTL for CD (*qCDs3*.*2*), LL (*qLLs3*.*1*), and LW (*qLWs3*.*1*) overlapped. The second region (22.35–49.45 cM) carried QTL for LL (*qLLs3*.*2*) and LW (*qLWs3*.*2*) (Table 2, Fig 3). Interestingly, we also found two previously reported [10] drought-related QTL located in this region, including a QTL identified for leaf relative water content (RWC) (RWC-S3.1, 31.13–32.44 cM) and a QTL for percent green cover (GC) (GC-S3.1, 32.69–33.10 cM) (Fig 3).

## Identification of candidate genes

Flanking sequences for SNP markers were used to search for candidate genes within QTL intervals. After gene function annotation, nine candidate genes were identified associated with drought tolerance response and regulation of plant growth and development (Table 3). These genes include two drought stress response genes: E3 ubiquitin-protein ligase (PUB23) and Beta-amylase 1 (BAM1); two root growth regulation related genes: LRR receptor-like serine/threonine-protein kinase (GSO1) and Auxin-responsive protein IAA12; two genes involved in stomatal movement regulation: root phototropism protein 2 (RPT2) and periodic tryptophan protein 2 (PWP2); and three genes involved in regulation of plant growth and development: Gibberellin 2-beta-dioxygenase 3 (GA2OX3), F-box/LRR-repeat protein 17 (FBL17) and S-adenosylmethionine decarboxylase proenzyme (SAMDC1) (Table 3).

**Table 3:** Identification of candidate genes within QTL intervals associated with drought tolerance and plant morphology in St. Augustinegrass.

Marker	QTL	Orthologous gene	Biological function
SNP27941	*qLLs3*.*1*	Root phototropism protein 2 (RPT2)	Stomata opening regulation [16]
SNP31468	*qLLs3*.*2*	Periodic tryptophan protein 2 (PWP2)	Stomatal movement regulation [17]
SNP51461	*qLLs3*.*2/qLWs3*.*2*	LRR receptor-like serine/threonine-protein kinase (GSO1)	Root growth regulation [18]
SNP46773	*qLLs3*.*1/qLWs3*.*1/qLWs3*.*2*	Gibberellin 2-beta-dioxygenase 3 (GA2OX3)	Plant architecture regulation [19]
SNP11556	*qLLs3*.*1/qCDs3*.*2*	E3 ubiquitin-protein ligase (PUB23)	Water stress response [20]
SNP27987	*qCDr9*.*1*	Beta-amylase 1 (BAM1)	Cold and drought stress responses [21]
SNP32817	*qCDs9*.*1*	F-box/LRR-repeat protein 17 (FBL17)	Regulation of shoot system morphogenesis [22]
SNP39736	*qCDs9*.*1*	Auxin-responsive protein IAA12	Primary root initiation [23]
SNP5205	*qSGOr1*.*1*	S-adenosylmethionine decarboxylase proenzyme (SAMDC1)	Plant embryogenesis, growth and development [24]

## Discussion

In the present study, we expanded on the available genomic and molecular information for St. Augustinegrass, one of the most important warm-season turfgrasses in the U.S., by taking advantage of high-density genetic maps and multi-environment experiments to conduct QTL analysis for morphological traits related to water usage. Currently, the application of molecular breeding for most turfgrass species is still in development, especially for St. Augustinegrass. Due to the absence of a reference genome in St. Augustinegrass, it often leads to mis-assembly and false positive SNP identification. In addition, the outcrossing nature of the species results in high levels of heterozygosity [25, 26]. With the enormous advancements in next-generation sequencing technology, high-throughput SNP markers were identified, and high-density genetic maps were developed in St. Augustinegrass more recently [9]. To date, QTL analyses have been reported for turf quality-related traits, freeze tolerance, drought tolerance, and gray leaf spot resistance in this species [9, 10, 11, 27]. However, we noticed that QTL for some traits not only influenced by genetic background, but also heavily affected by environment. Even though the same population was tested in this study and our previous report [9], there are only two common QTL region (*qCDs8*.*1 and qCDs8*.*2*) identified for canopy density in both studies, which showing significant environmental variable between different experimental trials. QTL with more environmental stability need to be validated in future prior to be applied in marker-assisted selection for St. Augustinegrass improvement.

In turfgrass, drought tolerance may be improved by possessing deep and extensive root systems that increase water uptake and morphological and physiological traits that reduce water loss. Water use is usually affected by water loss through shoot/leaf transpiration and soil evaporation, which are considered to be associated with shoot and canopy characteristics. Kim and Beard reported that turfgrass species showing prostrate shoot growth habit typically have lower water use rates than grasses with an upright growth habit [6]. Significant water use variation has been reported among different turfgrass species and cultivars, including St. Augustinegrass, tall fescue, bermudagrass, zoysiagrass, seashore paspalum, and centipedegrass [6]. Among them, St. Augustinegrass exhibited a medium-low water use rate [6]. Kim and Beard suggested that this might be due to low canopy resistance and high leaf area, which in turn result from St. Augustinegrass’ low shoot density, intermediate leaf orientation, wide leaf, and medium vertical leaf extension rate [6]. In the present study, we also observed a wide range of variation among the progeny for leaf width and length, canopy density, and shoot growth orientation (Table 1). In our previous study, this population (Raleigh x Seville) was reported to exhibit significant segregation for drought tolerance-related traits [10], which prompted us to further examine the correlation between morphological traits and drought-related traits. In general, LW and LL showed negative correlation with drought-related traits, while CD and SGO showed positive correlation (Fig 2). These results verified that higher LW and LL could enlarge the total leaf area and result in an increase in transpiration surface, which probably could increase water loss from transpiration. Meanwhile, higher canopy density and prostrate shoot growth orientation potentially contributed to high canopy resistance to water evaporation from soil.

Although the correlation between morphological traits and water use rate has been well described as above, the genetic basis of these traits has rarely been studied in turfgrass. The correlation between morphological and drought-related traits was further supported by the colocalization of QTL for those traits. In the present study, two previously reported drought-related QTL, one for leaf RWC and one for GC, were found to overlap with QTL for LL and LW on linkage group SLG3 (Fig 3) [10]. Although we did not detect overlap between drought QTL and CD and SGO QTL, we found overlapping QTL for SGO and LL on RLG1, and overlapping QTL for CD, LL, and LW on SLG3 (Table 2, Fig 3). These findings suggest that the genetic basis of leaf/canopy traits and drought related traits are partially overlapping in St. Augustinegrass. Within turfgrass species, QTL for morphological traits were also reported in bermudagrass and perennial ryegrass [28, 29]. QTL for drought tolerance were reported in bentgrass and St. Augustinegrass [10, 30, 31]. Currently, phenotypic recurrent selection has been successful in improving desirable traits in most turfgrass species, including St. Augustinegrass. QTL analyses suggest that molecular breeding methods, such as marker assisted selection, exhibit potential to be used in St. Augustinegrass breeding. The overlapping QTL identified in this study especially represent potential candidates for fine mapping and for further use in St. Augustinegrass improvement after validation.

We further investigated candidate genes within QTL intervals that potentially control drought tolerance and related morphological traits. Three candidate genes were found to be related to the regulation of plant growth and development. Gibberellin 2-beta-dioxygenase (GA2oxs) could regulate plant growth by inactivating endogenous bioactive gibberellins (GAs). Lo et al. (2008) reported that overexpression of GA2oxs in rice could results in semidwarfism, increased root systems and higher tiller numbers [19]. A F-box/LRR-repeat protein encoding gene (D3) was identified in rice tillering dwarf mutants through map-based cloning and demonstrated play a crucial role in the regulation of rice shoot branching through strigolactones signal pathways [22, 32]. S-adenosylmethionine decarboxylase proenzymes (SAMDCs) were identified from *Arabidopsis* bushy and dwarf mutant, which were essential for plant embryogenesis [24]. There were two water stress response genes identified. Cho et al. (2008) reported two E3 ubiquitin ligases, PUB22 and PUB23, coordinately control a drought signaling pathway by ubiquitinating cytosolic RPN12a in *Arabidopsis* [20]. Maruyama et al. (2009) suggested that expression of *BAM1*, encoding starch-degrading enzyme beta-amylase, increased under dehydration conditions but decreased under cold conditions [21]. Additionally, there were two genes that might contribute to drought tolerance by maintenance of extensive and deep root systems. A *GSO1* gene was identified in the interval of *qLLs3*.*2* and *qLWs3*.*2*, which controls primary root growth by modulating sucrose response [18]. It is interesting that our previous study also identified a *GSO1* gene in the interval of drought-related QTL *RWC-S3*.*1* [10], which was found to overlap with *qLLs3*.*2* and *qLWs3*.*2* in the current study. Further study will be needed to determine if the same gene is present within these QTL intervals. In addition, root phototropism protein 2 (RPT2) and periodic tryptophan protein 2 (PWP2) were suggested to be involved in the regulation of stomata opening and movement [16, 17], which is also a primary response to water balance in plants. However, due to the limitation of the population size, the mapped QTL and identified candidate genes are still preliminary, fine mapping will be conducted to validate in larger populations. In addition, further study of candidate genes is needed to determine their function and potential to be used to improve water use rate in St. Augustinegrass.

## Conclusions

In this study, we reported the genetic basis of complex morphological traits in St. Augustinegrass. QTL analysis revealed seven QTL for CD, four for LL, five for LW, and two for SGO under both single environment and across environments analysis. In addition, three overlapping QTL regions were identified on RLG1 and SLG3, and one of them on SLG3 overlapped with two previously reported drought-related QTL. Several candidate genes were identified within these QTL intervals that are involved in the regulation of plant development, stress response, root and stomata systems. This is the first report of QTL controlling morphological traits that potentially related to drought tolerance in St. Augustinegrass. Further fine mapping, QTL validation, and candidate gene identification will contribute to our understanding of the genetic control of morphological development in St. Augustinegrass. The putative QTL developed in this study have potential value to be utilized in St. Augustinegrass breeding programs through marker-assisted breeding.

## Supporting information

S1 File Genotypic and phenotypic data used for QTL mapping including genotype loci for hybrids, marker names and positions, and phenotypic data of morphological triats in the Raleigh x Seville population. (XLSX)

## Abbreviation

BLUP: Best linear unbiased predications
CD: Canopy density
ET: Evapotranspiration
GC: Percent green cover
GH: Greenhouses
IM: Interval mapping
LW: Leaf blade width
LL: Leaf blade length
MQM: Multiple QTL method
NDVI: Normalized difference vegetative index
NTEP: National Turfgrass Evaluation Program
QTL: Quantitative trait loci
RWC: Leaf relative water content
SGO: Shoot growth orientation
SNP: Single nucleotide polymorphism
SRS: Sandhills Research Station
TFL: Lake Wheeler Turf Field Laboratory
